# Prostate Cancer Biomarkers with a Focus on Galectin-3: Emerging Clinical and Therapeutic Implications

**DOI:** 10.3390/curroncol33050280

**Published:** 2026-05-09

**Authors:** Hiba Narvel, Mohammad Arfat Ganiyani, Adnan Gulam Nabi, Aman Goyal, Rohan Garje, Sanjay Srinivasan, Hafiz Ahmed, Deepak Kilari

**Affiliations:** 1 Department of Hematology and Oncology, Medical College of Wisconsin, Milwaukee, WI 53226, USA; 2 Department of Hematology and Oncology, Miami Cancer Institute, Baptist Health South Florida, Miami, FL 33176, USA; mohammadarfat.ganiyani@baptisthealth.net (M.A.G.);; 3 Pravara Institute of Medical Sciences, Loni 413736, India; 4 Department of Internal Medicine, Cleveland Clinic Foundation, Cleveland, OH 44195, USA; 5 GlycoMantra Inc., Baltimore, MD 21227, USA; sanjay@glycomantra.com (S.S.);

**Keywords:** prostate cancer, galectin-3 (Gal-3), precision oncology, biomarker, tumor microenvironment, therapeutic targeting

## Abstract

Prostate cancer (PCa) management has evolved with biomarker-driven strategies, yet biological heterogeneity, adaptive resistance, and an immunosuppressive microenvironment limit their efficacy. Galectin-3 (Gal-3) has emerged as a central node in PCa pathobiology, influencing tumor survival, metastasis, and immune escape. This review comprehensively reviews Gal-3’s dual role as a biomarker and a therapeutic target. We first delineate the limitations of the current diagnostic, prognostic, and predictive biomarkers in PCa, establishing the unmet need. We then elucidate the multifunctional biology of Gal-3, detailing its compartment-specific roles in anti-apoptosis, angiogenesis, epithelial-to-mesenchymal transition, and, notably, its function as a master regulator of immunosuppression. The interaction between Gal-3 and prostate-specific antigen (PSA) is explored as a key regulatory interface. Furthermore, we catalog and analyze emerging Gal-3-targeted therapies, emphasizing their rationale for combination with immune checkpoint blockade to reverse therapeutic resistance. Finally, we outline a translational roadmap, advocating for standardized Gal-3 biomarker assays and biomarker-enriched clinical trials. Integrating Gal-3 into the PCa precision medicine toolkit offers a novel strategy to address heterogeneity and improve therapeutic durability.

## Simple Summary

Prostate cancer is the most common cancer in men in the United States. While in some patients prostate cancer grows slowly and may never cause harm, in others, cancer can be aggressive and life-threatening. A major challenge is distinguishing between them early and choosing the right option for each person. Doctors currently rely on tests like PSA and genetic profiling, but these tools are imperfect, often expensive, and fail to predict which cancers will behave aggressively or respond to treatment. This review focuses on galectin-3, a protein that plays an important role in how prostate cancer grows, spreads, and evades the immune system. Galectin-3 can help cancer cells survive, travel to bones, and resist standard treatments. Importantly, galectin-3 can be measured in blood or tumor tissue, making it a potential biomarker. Overall, galectin-3 represents a promising new approach to aid in the care of prostate cancer.

## 1. Introduction

Prostate cancer (PCa) is the most commonly diagnosed cancer in men over the age of 60 and the second leading cause of cancer-related death in the United States (U.S.) [1]. The American Cancer Society projects approximately 333,830 new cases and 36,320 deaths from PCa in 2026 in the U.S. [2]. PCa is a biologically heterogeneous disease with varied outcomes ranging from indolent to rapidly lethal, making the identification and use of robust biomarkers to guide prognosis and care critical. Historically, prostate-specific antigen (PSA) has been the foundational biomarker for PCa detection and disease monitoring, yet its lack of disease specificity and sensitivity has spurred a search for more precise biomarkers [3]. The advent of advanced genomic and proteomic technologies has revolutionized our understanding of PCa biology, leading to the identification of a new generation of biomarkers [4]. Biomarkers in PCa have evolved to address distinct clinical needs across the disease continuum. Diagnostic biomarkers, such as the prostate health index (Beckman Coulter, Inc., Brea, CA, USA) (PHI), 4Kscore (OPKO Health, Inc., Miami, FL, USA), and SelectMDx (MDxHealth, Inc. Irvine, CA, USA), aim to improve specificity in initial detection, thereby reducing unnecessary biopsies in men with elevated PSA [5,6]. Following diagnosis, prognostic biomarkers (e.g., Decipher Veracyte, Inc., South San Francisco, CA, USA, Prolaris Myriad Genetics, Inc., Salt Lake City, UT, USA, Oncotype DX: Genomic Prostate Score Exact Sciences Corp., Madison, WI, USA) provide risk stratification by quantifying the biologic aggressiveness of the tumor, informing decisions regarding active surveillance versus definitive treatment [7,8]. Finally, predictive/therapeutic biomarkers guide precision therapy, especially in advanced disease. These include alterations in homologous recombination repair genes (e.g., BRCA1/2), which indicate susceptibility to poly (ADP-ribose) polymerase (PARP) inhibitors, as well as high tumor mutational burden (TMB) or microsatellite instability (MSI)/mismatch repair deficiency (dMMR), which can identify candidates for immune checkpoint inhibition [9,10,11]. Prostate-specific membrane antigen (PSMA)-PET scan has emerged as a promising theranostic tool with better sensitivity and specificity to stratify patients with metastatic prostate cancer who test positive to radiolabeled PSMA ligands such as 68Ga-PSMA-11a and 18F-DCFPyL on the tumor surface [12]. Despite this progress, several limitations persist in current PCa biomarkers.

PCa remains constrained by pronounced intra- and inter-tumoral heterogeneity [13], as well as by the relatively low prevalence of actionable genomic alterations with FDA-approved targeted therapies compared with other malignancies [14,15].

PSA is not a cancer-specific protein, and its expression is paradoxically suppressed by androgen signaling in aggressive, androgen-independent disease [16]. A fundamental limitation of PSA is its inherent biological complexity as an androgen-regulated protein. Notably, in advanced androgen-independent disease, androgen signaling can suppress PSA expression, decoupling serum PSA levels from true disease burden, highlighting its inadequacy as a stand-alone biomarker for disease monitoring across all stages [11]. Galectin-3 (Gal-3) is a β-galactoside-binding lectin with intracellular and extracellular functions that influence survival signaling, adhesion and migration, angiogenesis, epithelial-to-mesenchymal transition, and immune regulation [17,18]. Across solid tumors, Gal-3 impairs T-cell function and remodels myeloid populations, which supports an immunosuppressive microenvironment by directly inhibiting CD8^+^ and CD4^+^ T-cell activation and cytotoxicity, skewing macrophages and myeloid-derived suppressor cells (MDSCs) toward suppressive phenotypes, impairing dendritic cell-mediated priming, and remodeling the extracellular matrix to physically exclude immune cells [19,20,21]. In PCa, Gal-3 has been linked to metastatic behavior and treatment resistance, and it is measurable in tissue and in circulation [22]. Gal-3 is measured by immunohistochemistry (IHC), Western blot, or reverse transcription polymerase chain reaction (RT-PCR; mRNA expression levels of the *LGALS3* gene) in tissue, and by enzyme-linked immunosorbent assay (ELISA) or a fluorescence immunological assay (TRFIA) in blood; expression of Gal-3 is elevated in both tissue and blood in various cancers. Gal-3 is sequestered within the intracellular environment, where its localized density can be high. However, the correlation between tissue expression and circulating levels is complex; tissue levels provide insight into localized cellular behavior, while blood levels serve as a systemic indicator of the total biological burden and tumor inflammation [23,24].

Accordingly, this review (i) delineates the current landscape of precision diagnostics in PCa and the biological and systemic limitations that constrain their impact; (ii) evaluates how Gal-3 could be a biomarker and help overcome some of these limitations; and (iii) synthesizes the evidence for therapeutic Gal-3 inhibition in the future.

## 2. Biomarkers in PCa

### 2.1. Definitions

The National Cancer Institute defines a biomarker as a measurable molecule in blood, other body fluids, or tissues that reflects normal or abnormal biologic processes. Biomarkers are essential for selecting therapy, designing trials, and assessing outcomes.

For PCa, a biomarker is any objective molecular, genomic, or cellular signature that provides actionable information distinct from traditional clinicopathologic parameters. Its utility is defined by the clinical question it addresses: diagnostic, prognostic, and predictive (therapeutic) biomarkers identify molecular alterations within the tumor that confer sensitivity or resistance to a specific targeted agent. The evolution of PCa biomarkers reflects the field’s progression from managing a homogeneous entity to orchestrating precision interventions for a molecularly heterogeneous group of diseases.

### 2.2. Current Landscape of Biomarkers Used in PCa

The first biomarker used for PCa was prostate acid phosphatase (PAP), which was replaced by PSA in the 1980s because PAP was a late-stage marker detectable only in advanced, incurable disease, whereas PSA enabled the early detection of potentially curable disease [14]. PSA, also known as kallikrein-3, is a glycoprotein (serine protease) encoded by the *KLK3* gene and secreted in small quantities in normal prostatic tissue but is elevated in PCa [25]. PSA continues to play an important role in PCa diagnosis and management, but it has several limitations, such as the fact that it is organ-specific but not always disease-specific [26]. There is a quest for biomarkers that can distinguish between aggressive and indolent tumors, thereby leading to better disease assessment and treatment strategies. Table 1 below provides an overview of emerging liquid-based biomarkers, such as circulating tumor cells (CTCs) and cell-free DNA (cfDNA), that offer non-invasive alternatives for early detection and disease assessment. Early reductions in cfDNA concentration or circulating tumor DNA (ctDNA) percentage (ctDNA%) within 4–8 weeks of initiating systemic therapy are associated with longer progression-free and overall survival. Conversely, increases in ctDNA% at 12 weeks may indicate a higher risk of early disease progression, supporting ctDNA kinetics as an on-treatment biomarker to inform response-adapted decisions [27]. Additionally, urinary biomarkers such as PCA3 and TMPRSS2-ERG fusion transcripts enhance diagnostic accuracy when used alongside PSA [28].

After diagnosis, risk stratification is crucial for treatment decisions. Genomic classifiers like Decipher and Oncotype DX Prostate analyze tumor RNA expression to predict aggressiveness, metastasis risk, and PCa-specific mortality, aiding in personalized therapy selection [7,8].

Exosome-based biomarkers and microRNA signatures show promise in predicting metastases and prognoses in PCa patients [45]. Additionally, artificial intelligence (AI)-driven biomarker panels are being developed to integrate diverse data for improved decision-making. Artera AI Prostate, a multimodal artificial intelligence (MMAI) prognostic biomarker, was developed and validated using data from randomized trials in localized PCa to prognosticate multiple clinically relevant endpoints. The MMAI prognostic biomarker was validated using data from men with high-risk PCa across six Phase 3 randomized trials and was independently prognostic relative to standard clinical and pathologic variables [46]. Pathogenic alterations in homologous recombination repair genes, especially BRCA1 and BRCA2, identify patients likely to benefit from poly (ADP-ribose) polymerase inhibitor-based regimens in metastatic disease, so coordinated germline and somatic testing has become routine for selection and for family counseling where appropriate. Germline testing is recommended because a significant proportion (up to ~12%) harbor actionable hereditary mutations, regardless of family history or cancer stage [47]. PSMA expression on PET serves as a true theranostic selector for lutetium-177 PSMA radioligand therapy, aligning target presence with the mechanism of action [48]. SUV (standardized uptake value) mean reflects the average expression level of PSMA across a tumor volume and is generally associated with more aggressive disease biology and worse clinical outcomes (shorter time to progression, shorter survival). Androgen-receptor pathway dependence and acquired resistance, including splice variants such as AR-V7, guide choices between continued androgen-receptor pathway inhibition and taxanes in the right clinical context [49]. Taken together, an integrated biomarker ecosystem that starts with high diagnostic yield, robust prognostic stratification, and directs therapy selection now underpins contemporary precision care in PCa.

### 2.3. Limitations of Current Biomarkers

Despite these advances, the path from current biomarkers to personalized, effective treatment is fraught with challenges that limit their utility and accessibility. Unlike some other cancers with a single dominant driver mutation, PCa is characterized by a high degree of biological complexity that challenges a straightforward biomarker-driven approach [13].

Current diagnostic biomarkers for PCa are limited by biological heterogeneity, insufficient specificity for clinically significant disease, assay variability, and incomplete population-level validation, resulting in persistent overdiagnosis, underdetection of aggressive tumors, and uneven clinical adoption. The most commonly used serum PSA test has about 90% sensitivity (true positives) and between 9% and 33% specificity (false positives). With increased use of PSA testing, many individuals experienced adverse effects and unnecessary procedures because their PSA values were elevated secondary to benign conditions. The biggest unmet need is a biomarker that accurately identifies truly aggressive cancers at screening, avoiding unnecessary interventions for slow-growing cancers.

Current prognostic biomarkers in PCa improve population-level risk stratification but are limited by tumor heterogeneity, static sampling, reliance on surrogate endpoints (such as biochemical recurrence-free or metastases-free survival rather than overall or cancer-specific survival), and incomplete linkage to treatment responsiveness and long-term survival [50]. They are also often therapy-agnostic, not translating into actionable therapeutic guidance, since they do not reliably predict benefit from treatment intensification or interaction with systemic therapies. As a result, they refine risk estimates without fully capturing the dynamic biology that determines lethal disease.

Most prognostic or genetic biomarkers in prostate cancer are single-time-point measurements that fail to capture dynamic tumor evolution under therapeutic pressure, such as clonal selection under androgen deprivation therapy (ADT), the emergence of AR-independent or neuroendocrine phenotypes, or treatment-induced genomic instability. While these biomarkers have enabled precision therapy for certain molecular subsets, their clinical utility remains limited by tumor heterogeneity, static sampling in a progressive disease, insufficient accounting for non-genetic drivers of lethality, and inadequate validation against long-term outcomes. Consequently, baseline genomic profiles can become biologically obsolete as the disease advances.

Serial genomic monitoring is feasible (ctDNA) but not yet standardized or universally sensitive. Additionally, reliable predictive and pharmacodynamic markers are limited for rational immunotherapy combinations, including assays that indicate when to combine or sequence therapies and that report early on-treatment benefit or futility [51]. Moreover, the metastatic niche, particularly bone, is not adequately addressed by targeted strategies that integrate tumor, stromal, and immune biology, and there are no validated biomarkers that reflect the interplay among adhesion, migration, angiogenesis, epithelial-to-mesenchymal transition, and osteoclastogenesis [52].

#### 2.3.1. Intra- and Inter-Tumor Heterogeneity

A primary obstacle to targeted therapeutic approaches for PCa lies in its inherent genomic complexity. Next-generation sequencing (NGS) has illuminated a diverse molecular landscape, yet translating this information into actionable clinical strategies remains difficult. A notable study found that NGS was ordered for PCa patients at a rate 10 times lower than for lung cancer patients, suggesting minimal clinical adoption [53]. The data from these genomic analyses highlight two critical issues. First, there is a high degree of both inter- and intra-tumoral heterogeneity. In a study of 67 PCa patients, no two individuals had identical molecular profiles, with a median of three unique genomic alterations per patient. This level of patient-to-patient variability complicates the development of universal biomarker-driven therapies. Second, while a large percentage of patients (84% in one cohort) have at least one potentially actionable alteration, the overall prevalence of highly “druggable” and FDA-approved targets common in other cancers, such as BRAF or ERBB2, is low [54,55,56]. This creates a fundamental problem: a patient may undergo expensive genomic profiling and discover multiple mutations, but a corresponding FDA-approved targeted therapy may not yet exist for any of them. This “low prevalence” problem is a central roadblock that limits the number of patients who can benefit from a personalized treatment approach based on rare, specific mutations.

The low clinical utility of genomic testing for many patients disincentivizes providers from adopting it widely. This creates a self-perpetuating cycle in which limited therapeutic options stifle demand for diagnostic testing, thereby impeding the very development of a robust precision medicine ecosystem.

#### 2.3.2. Therapeutic Evolution and Resistance in PCa

A central limitation of precision medicine in PCa is the almost inevitable development of therapeutic resistance, which remains a major cause of treatment failure and patient mortality. Most patients with advanced disease who initially respond to ADT eventually progress to a castration-resistant prostate cancer (CRPC) state within 1 to 2 years [57]. This resistance is not a simple phenomenon, but a complex biological adaptation driven primarily by the reactivation of the androgen receptor (AR) signaling pathway.

The androgen receptor (AR) is not only a central driver of prostate cancer (PCa) growth but also a key mediator of therapeutic resistance, particularly to androgen-deprivation therapy (ADT) and next-generation AR-targeted agents. Resistance can arise through multiple mechanisms, including AR gene mutations or amplification, ligand-independent activation, production of constitutively active splice variants (e.g., AR-V7), and cross-talk with alternative signaling pathways such as HER2 and MAPK. Collectively, these mechanisms enable tumor survival and progression to castration-resistant prostate cancer (CRPC) by reactivating AR signaling, enhancing DNA repair capacity, and promoting cellular plasticity, making AR targeting both complex and essential [58].

Moreover, epigenetic changes such as DNA methylation, particularly hypomethylation, and changes in promoter regions of AR target genes can lead to increased gene expression and AR reactivation even with ADT. Histone modification can affect AR transcriptional activity, while non-coding RNAs can regulate AR expression. Studies have shown that AR-V7 expression increases with progression to CRPC, and a series of recent studies found that AR-V7 expression in CTCs is associated with Abiraterone/Enzalutamide resistance [59].

A subset of prostate cancers evades AR-targeted therapies through the development of lineage plasticity [42,60]. This is associated with loss of AR or AR signaling, frequent RB1/TP53 loss-of-function, and activation of alternative lineage programs, including the neuronal, neuroendocrine, stem-like, and developmental pathways. These alterations reveal vulnerabilities, such as dependence on DNA repair (exploited by PARP/ATR inhibitors) or reliance on SOX2/EZH2 pathways (targeted by epigenetic drugs). In essence, the concurrent loss of PTEN, TP53, and RB1 is a genetic hallmark of prostate cancer’s capacity to transform into aggressive, non-adenocarcinoma forms, making them vital biomarkers for risk stratification and treatment selection [60].

A core principle of precision medicine is the ability to match a patient’s specific genomic alteration with a corresponding targeted therapy. In PCa, the efficacy of this model is significantly constrained by the limited prevalence of targetable genomic alterations compared to other cancer types. For instance, a study of patients who underwent NGS found that while 84% had at least one potentially actionable alteration, a minority harbored alterations—such as those in BRAF, NTRK, or mismatch repair genes—that are highly responsive to targeted therapies in other cancers [13,38].

#### 2.3.3. Gal-3 in PCa and Its Role in Tumor Proliferation, Angiogenesis, and Metastasis

Gal-3 is expressed in a wide range of malignancies and plays a well-established role in tumor progression and metastasis [55,61,62,63,64]. Across the gastric, liver, lung, bladder, and head and neck cancers, Gal-3 expression is significantly increased compared with normal tissues and correlates with disease progression and metastatic potential. Functionally, Gal-3 is a pleiotropic regulator of cancer biology, influencing apoptosis resistance, metastatic dissemination, immune surveillance, molecular trafficking, mRNA splicing, transcriptional regulation, and inflammatory signaling [65].

Importantly, Gal-3 function is highly dependent on cellular localization, a property central to understanding its context-specific role in PCa. In the cytoplasm, Gal-3 modulates the AKT signaling pathway, promoting cell survival in part through the inhibition of transforming growth factor-β (TGF-β)-mediated apoptosis. In the nucleus, Gal-3 regulates pro-mRNA splicing and transcriptional programs. In the extracellular compartment, Gal-3 promotes integrin-mediated cell adhesion, migration, endothelial cell morphogenesis, and angiogenesis—processes critical for cancer progression and metastatic spread [66]. Integrin signaling downstream of extracellular Gal-3 frequently involves activation of focal adhesion kinase (FAK) and Src family kinases, driving cell survival and migration, with the additional activation of MAPK/ERK pathways that regulate proliferation and differentiation [67].

The extracellular and microenvironmental functions of Gal-3 are particularly relevant in the context of PCa bone metastasis. Gal-3 facilitates prostate cancer cell adhesion to myosin-2A, a regulator of osteoclastogenesis, thereby promoting increased bone remodeling and turnover within the metastatic niche [57]. At the intracellular level, membrane and endoplasmic reticulum-associated signaling cascades in androgen-independent PCa cells upregulate Gal-3 and stabilize non-phosphorylated β-catenin. Gal-3 directly binds β-catenin/TCF4 complexes, enhancing TCF4 transcriptional activity and driving proliferation, migration, and invasion. Similar Gal-3-mediated inhibition of AKT/GSK-3β signaling has been shown to reduce β-catenin degradation and promote nuclear translocation in colon cancer, underscoring the conserved role of Gal-3 as a regulator of Wnt/β-catenin signaling across malignancies [68].

Gal-3 also contributes to metastatic competence through induction of epithelial-to-mesenchymal transition (EMT). Gal-3 cooperates with TGF-β signaling to downregulate epithelial adhesion programs and promote cellular plasticity, enabling tumor cells to migrate and invade distant tissues [54]. Beyond tumor-intrinsic effects, Gal-3 is a potent mediator of immune evasion. It suppresses tumor-reactive T-cell expansion, induces CD8^+^ T-cell apoptosis, impairs T-cell receptor clustering, and shifts macrophage polarization from pro-inflammatory M1 states toward immunosuppressive M2 phenotypes, collectively promoting tumor progression and metastasis [54].

Despite this strong pro-tumor biology, reported patterns of Gal-3 expression across PCa disease stages appear heterogeneous. Some studies describe reduced Gal-3 expression in epithelial tumor cells during progression from localized to advanced disease [55], whereas others emphasize Gal-3 re-expression or elevation in metastatic and castration-resistant settings [69,70,71]. These observations are not contradictory but instead reflect stage-specific, compartment-dependent biology and differences in analytic context.

Gal-3’s pro-tumor activity across malignancies is fundamentally driven by its potent anti-apoptotic function and its role as a central node for microenvironmental signaling. Intracellularly, Gal-3 directly inhibits mitochondrial apoptosis by sequestering pro-apoptotic proteins and activating survival pathways [72]. Upon secretion, Gal-3 binds cell-surface glycoproteins to activate proliferative and invasive programs while simultaneously orchestrating immune evasion by suppressing cytotoxic T-cell function [72]. This compartment-dependent duality makes Gal-3 a central regulator of tumor progression and a compelling, multifaceted therapeutic target [69]. Notably, Gal-3 predominantly exists in a cleaved form with higher affinity for endothelial cells than full-length Gal-3, further enhancing angiogenesis and metastatic potential [70].

In PCa specifically, Gal-3 exerts profound effects on the tumor microenvironment (TME). Gal-3 produced by tumor cells and stromal components suppresses immune surveillance by recruiting and activating myeloid-derived suppressor cells (MDSCs) and regulatory T cells (Tregs) while limiting interferon-γ penetration into the TME, leading to reduced infiltration of cytotoxic T lymphocytes and promoting immune escape [71,73]. These observations have led to the conceptualization of Gal-3 as a dominant immunosuppressive checkpoint particularly relevant in advanced PCa.

Gal-3 also participates directly in AR pathway adaptation. Experimental studies demonstrate high Gal-3 expression in metastatic, androgen-independent PCa cell lines (DU145, PC3) but minimal or absent expression in androgen-dependent LNCaP cells [68,74], implicating Gal-3 in the transition from androgen dependence to castration-resistant disease [75]. In this context, Gal-3 also emerges as a potential diagnostic and prognostic biomarker, particularly as a complement to PSA, which is limited by a lack of cancer specificity and androgen-dependence [76]. Intriguingly, Gal-3 is a substrate for PSA, establishing a direct biochemical and regulatory link between these two proteins [68,77]. PSA-mediated cleavage can alter Gal-3 structure and function, potentially modulating its pro-apoptotic and cell-adhesive properties. Loss of this regulatory interaction in advanced, androgen-independent disease—where PSA expression is often dysregulated—may permit the accumulation of intact, biologically active Gal-3, contributing to aggressive, treatment-resistant phenotypes. Despite its strong mechanistic relevance, Galectin-3 has important limitations as a biomarker, most notably its lack of cancer-type specificity. Gal-3 is expressed in a broad range of solid tumors—including gastrointestinal, breast, lung, and bladder cancers—as well as in non-malignant inflammatory and fibrotic conditions, which may confound its use as a prostate-specific diagnostic marker. Accordingly, elevated tissue or circulating Gal-3 levels should not be considered disease-specific in isolation. This broad expression profile may limit its utility as a standalone diagnostic marker. In this context, the clinical value of Gal-3 may be better realized when used in combination with established prostate-specific biomarkers or within multi-analyte panels. Furthermore, emerging evidence suggests that differences in expression patterns, subcellular localization, and circulating levels may provide additional discriminatory value, although these aspects require further validation. Importantly, despite growing interest in Galectin-3 as a tissue-based and circulating biomarker, its clinical translation is limited by a lack of assay standardization. Current studies employ heterogeneous detection platforms—including immunohistochemistry, ELISA, time-resolved fluorescence immunoassays, and mRNA-based methods—with variable antibodies, thresholds, and reporting units, complicating cross-study comparisons and precluding the establishment of clinically validated cut-off values. Although Galectin-3 can be assessed in both tumor tissue and circulation, direct comparisons between tissue-based and circulating Gal-3 levels, and their relationship to dynamic biomarkers such as circulating tumor DNA (ctDNA) kinetics or quantitative PSMA-PET imaging parameters have not been systematically studied. While each of these biomarkers has demonstrated independent prognostic or predictive value in prostate cancer, the lack of integrated, cross-modal analyses currently limits conclusions regarding their inter-relationships. As such, the direct integration of Gal-3 with ctDNA dynamics or PSMA-PET metrics remains an important gap in the translational literature and represents a key area for future biomarker-enriched, multimodal studies.

Clinically, translational studies demonstrate that serum Gal-3 levels are consistently higher in patients with metastatic PCa compared with cancer-free controls, with comparable or superior discriminatory performance to PSA in selected cohorts. In this selected cohort, the AUC was 1.0 (based on the non-overlapping ranges of 0.10–0.43 vs. 0.00–0.06 ng/mL), which exceeds the standard historical performance of PSA for identifying metastasis, particularly in “false negative” PSA cases [78,79]. While some studies report a positive correlation between Gal-3 and PSA levels (rho = 0.446, *p* < 0.0001) [80], broader pan-cancer analyses confirm significantly elevated circulating Gal-3 in metastatic compared with localized disease [81].

Furthermore, composite indices integrating Gal-3 with PSMA expression correlate positively with Gleason score (r^2^ = 0.713, *p* < 0.001) and outperform either PSMA (r^2^ = 0.597, *p* < 0.001), or Gal-3 alone (r^2^ = 0.434, *p* < 0.001) in predicting tumor aggressiveness [22]. Collectively, these findings support a biphasic and compartmental model in which epithelial Gal-3 expression may be suppressed in early localized disease, while Gal-3 re-emerges as a dominant extracellular, microenvironmental, and circulating mediator in advanced and metastatic PCa.

## 3. Targeting Gal-3

**Figure 1:**
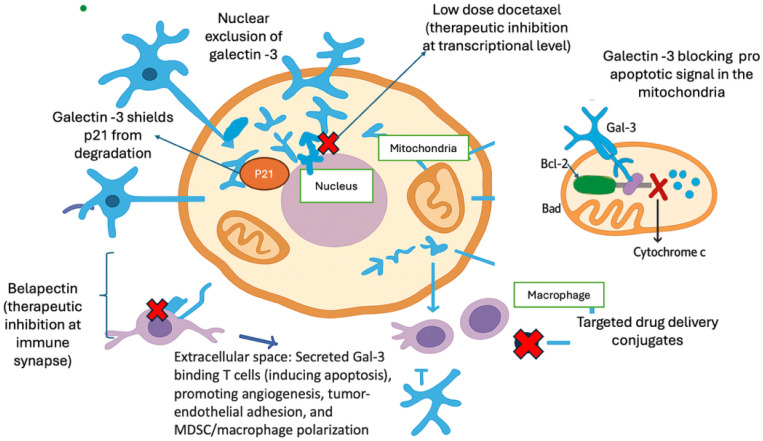
Compartment-specific functions of Galectin-3 and therapeutic targeting strategies in prostate cancer.

Targeting Gal-3 as a therapeutic strategy in cancer treatment has gained significant attention in recent years, owing to its critical role in cancer progression and metastasis [82]. In particular, blocking Gal-3’s interaction with immune cells may enhance anti-tumor immune response. This is strongly relevant in cancers with high levels of immunosuppressive cells, such as PCa. However, Gal-3 promotes tumor progression by binding to integrins and other extracellular ligands, further suggesting that anti-Gal-3 regimens could be crucial for disease management. Figure 1 depicts Galectin-3’s interactions and how it may be therapeutically targeted. Several approaches to targeting Gal-3 have been explored, ranging from small- and large-molecule inhibitors to monoclonal antibodies and carbohydrate-based antagonists.

### 3.1. Carbohydrate-Based Competitive Antagonists

These compounds function by directly occupying the carbohydrate-recognition domain (CRD) of Gal-3, blocking its interaction with natural glycoprotein ligands. This class includes chemically modified mono- or disaccharides structured around galactose, lactose, thiodigalactoside (TDG), and lactulose [82]. Notable examples are modified citrus pectin (MCP) and belectin (GR-MD-02), which are polysaccharide derivatives rich in galactose. By binding to Gal-3, they disrupt its pro-metastatic signaling, inhibiting cell adhesion, migration, and angiogenesis. Belapectin has been shown to modulate the tumor microenvironment by influencing myeloid-derived suppressor cells (MDSCs) and restoring T-cell-mediated cytotoxicity, thereby enhancing survival in immunocompetent prostate and breast cancers [28].

### 3.2. High-Affinity Small and Large Molecule Inhibitors

This approach utilizes synthetic, low-molecular-weight compounds, including TD139 (GB0139), GB1107, and GB1211, designed for high affinity and specificity to the Gal-3 CRD [17]. TD139 (GB0139) is a thiodigalactoside derivative and the most clinically advanced candidate, demonstrating potent inhibition of Gal-3-mediated angiogenesis and immune evasion [83]. Next-generation inhibitors like GB1107 and GB1211 exhibit strong binding and have shown promising preclinical activity in enhancing anti-tumor immunity. GB1107, for instance, increases CD8+ T-cell infiltration and synergizes with anti-PD-L1 therapy to boost cytotoxic effector molecules [83]. In addition, GB1107 also binds Gal-3 with high affinity and primarily enhances CD8+ T cell infiltration within the tumor microenvironment [56]. In one study, GB1107 was observed to potentiate the effects of a PD-L1 immune checkpoint inhibitor, increasing the expression of cytotoxic (IFN-gamma, granzyme B, perforin-1, Fas ligand) and apoptotic (cleaved caspase-3) effector molecules [84]. GB1211 (which shares a chemical template with GB1107) has shown efficacy in reversing Gal-3-mediated blockade of PD-1/PD-L1 in non-small cell lung cancer (NSCLC) [85]. GlycoMantra has developed a very high-affinity recombinant glycoprotein inhibitor of Gal-3 based on a few modifications of its patented drug TFD100 originally isolated from edible cod [82,86].

Large-molecule inhibitors such as modified citrus pectin (MCP) and GCS-100 have been evaluated for their roles in inhibiting Gal-3 [82]. Notably, MCP, which is abundant in galactose, binds directly to Gal-3 within PCa cells, blocking its interaction with extracellular carbohydrate substrates and antigens. This disruption inhibits downstream signaling pathways involved in cell migration, angiogenesis, and tumor progression. GCS-100 influences the immune response by inhibiting the interaction between Gal-3 and CD4+ and CD8+ tumor-infiltrating lymphocytes, boosting cytotoxicity, and restoring IFN-gamma secretion [46]. This is the same mechanism by which Gal-3 inhibitors can augment the effects of immune checkpoint blockade (ICB) against tumor cells.

### 3.3. Antibody-Based Neutralization

Monoclonal antibodies offer a highly specific mechanism to neutralize extracellular Gal-3. The antibody 14D11, for example, competes with lactose for the Gal-3 CRD [33]. This binding inhibits downstream oncogenic pathways (e.g., AKT and ERK phosphorylation) and reduces cancer cell invasion, prolonging survival in animal models [87]. Clinically, the efficacy of immune checkpoint inhibitors like anti-CTLA-4 has been correlated with the induction of endogenous anti-Gal-3 antibodies, suggesting that therapeutic antibody-mediated neutralization could directly inhibit tumorigenesis and augment cancer therapy [88].

## 4. Synergy with Immunotherapy: Overcoming an Immune Checkpoint

The most compelling therapeutic rationale for Gal-3 inhibition lies in its synergy with immune checkpoint blockade (ICB). Gal-3 acts as a soluble negative immune checkpoint by shielding tumor cells from T-cell attack and directly interfering with anti-PD-1 antibody binding. Inhibition of Gal-3 can thus reverse primary resistance to ICBs [45]. Transcriptomic analyses reveal that metastatic PCa frequently re-expresses high levels of Gal-3, identifying it as a key mediator of immunotherapy failure. Clinical proof-of-concept comes from a Phase I study combining belapectin with pembrolizumab in metastatic melanoma and head and neck cancer, which achieved objective response rates of 50% and 33%, respectively—substantially higher than pembrolizumab monotherapy [89]. These findings establish Gal-3 as both a biomarker of ICI resistance and a therapeutic co-target to “prime” otherwise unresponsive tumors. In PCa, low-dose docetaxel has been found to downregulate this Gal-3 checkpoint, providing a mechanistic basis for combination strategies.

In PCa, Gal-3 expression is re-established at advanced and metastatic stages, where it functions as the primary immune checkpoint driving immunotherapy failure [90]. Given the limited prevalence of actionable genomic alterations in PCa, Gal-3 inhibition represents an attractive new strategy, particularly in combination with ICIs.

## 5. Genetic and Protein-Based Strategies

Alternative strategies include the use of a genetically engineered, dominant-negative form of Gal-3 lacking the N-terminal domain [45]. This mutant competes with endogenous Gal-3 for carbohydrate binding sites, thereby inhibiting its canonical functions in cell motility and angiogenesis [70]. While primarily a research tool, this concept underscores the importance of the CRD and validates its inhibition as a therapeutic goal.

Table 2 below summarizes the evidence for Gal-3 inhibition in solid tumors [82,89,91,92,93].

## 6. Ongoing Trials Evaluating Combination Therapies with Galectin-3 Inhibitors

Galectin-3 (Gal-3) has emerged as a promising immunomodulatory target to overcome resistance to immune checkpoint inhibitors (ICIs), with several combination strategies under investigation. The oral Gal-3 inhibitor GB1211 (Galecto Biotech, Boston, MA, USA) was evaluated in the GALLANT-1 trial (NCT05240131), a Phase Ib/IIa study combining GB1211 with atezolizumab in first-line advanced NSCLC adenocarcinoma with PD-L1 ≥ 50%; while Part A dose-finding demonstrated an acceptable safety profile, the randomized Part B was not pursued as Galecto pivoted its strategic focus to liver fibrosis, though the company continues to supply GB1211 for investigator-initiated oncology studies [98]. A second trial, GALLANT-2 (NCT05913388), a Phase 2 randomized, placebo-controlled study of GB1211 plus pembrolizumab in metastatic melanoma and head and neck squamous cell carcinoma (HNSCC), remains actively recruiting. These efforts build upon encouraging Phase 1 data with the earlier Gal-3 inhibitor belapectin (GR-MD-02) combined with pembrolizumab (NCT02575404), which demonstrated objective response rates of 50% in melanoma (7/14 patients) and 33% in HNSCC (2/6 patients), with fewer immune-mediated adverse events than expected with pembrolizumab monotherapy [89]. Mechanistically, Gal-3 inhibition addresses multiple resistance pathways including T-cell exclusion, immune exhaustion, and immunosuppressive myeloid cell activation, with some Gal-3 inhibitor combinations receiving FDA Fast Track designations [99]. Beyond Gal-3, anti-galectin-9 antibodies (LYT-200) are also entering clinical evaluation in combination with ICIs and DNA damage response inhibitors. While no trials currently combine Gal-3 inhibitors with ^177^Lu-PSMA radioligand therapy, preclinical data demonstrate synergistic cytotoxicity when PSMA and Gal-3 are co-targeted in prostate cancer cell lines, suggesting a rational future combination strategy.

## 7. Conclusions

Precision oncology for PCa has progressed from single-analyte serum screening to a multilayered biomarker ecosystem spanning urine/serum assays, tumor genomics, and advanced molecular imaging, such as PSMA-PET. However, marked intra- and inter-tumoral heterogeneity, the relative scarcity of highly druggable alterations, and the microenvironmental and immune barriers that drive resistance continue to blunt the therapeutic benefit. Galectin-3 occupies a compelling intersection of tumor biology and immune regulation, with mechanistic links to adhesion signaling, epithelial-to-mesenchymal transition, angiogenesis, androgen-axis adaptation, and immune evasion.

Early translational data suggest that Gal-3 inhibition—particularly in combination with immune checkpoint blockade and AR-directed or radioligand therapies—may convert resistant phenotypes into responsive ones. Realizing this potential will require: (i) assay standardization with analytically validated cut-offs for tissue and circulating Gal-3; (ii) prospective, biomarker-enriched trials that test Gal-3 inhibitors with rational partners and include on-treatment pharmacodynamic readouts (ctDNA kinetics, immune signatures); and (iii) the integration of Gal-3 into composite indices with PSMA and genomic markers to refine patient selection. In summary, Galectin-3 represents a promising but still investigational biomarker and therapeutic target in prostate cancer. Realizing its clinical utility will depend on assay standardization, prospective validation, and integration into rational, biomarker-driven clinical trial designs.

Schematic representation of the context-dependent roles of Gal-3 across intracellular, nuclear, and tumor microenvironment compartments in prostate cancer. During disease progression, Gal-3 is increasingly excluded from the nucleus, accompanied by loss of tumor-suppressive nuclear functions. In the cytoplasm, Gal-3 promotes tumor cell survival by stabilizing p21 and inhibiting mitochondrial apoptosis through interaction with the Bcl-2/Bad axis, thereby preventing cytochrome c release. Extracellularly, secreted Gal-3 contributes to immune evasion by binding glycosylated receptors on T cells, inducing apoptosis, impairing immune synapse formation, and promoting angiogenesis, tumor–endothelial adhesion, and immunosuppressive macrophage and myeloid-derived suppressor cell polarization. Key therapeutic intervention points are indicated, including Gal-3 carbohydrate-recognition domain inhibitors, immune synapse blockade with belapectin, transcriptional downregulation of Gal-3 with low-dose docetaxel, and emerging Gal-3-directed drug delivery conjugates.

## Figures and Tables

**Table 1:** Overview of biomarkers for PCa.

A. Diagnostic Biomarkers					
Biomarker	Diagnostic or Prognostic; Sample Type	Main Purpose	Approval Status with Year of Approval	Indication	Limitation; Evidence Level (Based on NCCN Recommendation)
PSA (Prostate-Specific Antigen)	Diagnostic; Serum	Screening and monitoring; detects prostate tissue activity.	FDA approved (1994)	Initial screening and disease monitoring [29].	Limited disease specificity; elevated in benign prostatic hyperplasia and prostatitis. NCCN: Category 1
proPSA and prostate health index	Diagnostic (p2PSA/fPSA) × PSA½; Serum	Distinguish cancer from benign conditions, in PSA between 4–10 ng/dL	FDA approved (2012)	Significantly improved the predictive accuracy (31% increase in specificity over PSA; AUC 0.7 vs. 0.53) for detection of PCa [30,31].	Limited disease specificity; false positives in prostatitis/BPH; limited data at PSA > 10 ng/mL. NCCN: Category 2A
PCa antigen 3 (*PCA3* or *DD3*)	Diagnostic NAAT; urine	Ruling out PCa, in those suspected to have PCa based on PSA level and/or DRE and/or one or more negative biopsy results.	FDA approved (2012)	While PCA3 demonstrates lower sensitivity than serum PSA (57% vs. 92%), its specificity is significantly superior (85% vs. 16%) in the diagnostic ‘gray zone’ of PSA 4–10 ng/mL. Specifically, in patients with a previous negative biopsy, a PCA3 score > 35 is associated with a negative predictive value (NPV) of approximately 88% [32].	Limited by mRNA instability and racial variability in TMPRSS2: ERG fusion (lower in Asian/Black men) NCCN: No longer listed
SelectMDx	Diagnostic; Urine	mRNA test (HOXC6, DLX1) + clinical risk factors.	CE-marked	Pre-biopsy risk assessment for clinically significant PCa [33].	Limited validation in diverse populations; performance post-MRI is uncertain. NCCN: Category 2A
ExoDx Prostate (IntelliScore)	Diagnostic; Urine	Exosome gene expression (ERG, PCA3, SPDEF).	CLIA-validated (2016)	Pre-biopsy risk assessment, independent of PSA [34].	Cost considerations; long-term clinical utility and cost-effectiveness still under evaluation. NCCN: Category 2A
**B. Prognostic Biomarkers**					
**Biomarker**	**Diagnostic or Prognostic;** **Sample Type**	**Main Purpose**	**Approval Status**	**Indication**	**Limitation(s);** **Evidence Level (Based on NCCN Recommendation**
OncotypeDX Genomic Prostate Score	Prognostic; biopsy tissue	Predicts PCa-specific death post-TURP; GPS: Identifies indolent tumors for active surveillance.	CLIA-validated (2013)	Predicts adverse pathology, biochemical recurrence, and PCa-specific outcomes; helps identify indolent tumors suitable for active surveillance [8].	Limited utility in high-risk disease; requires adequate biopsy tissue; cost considerations. NCCN: Not specifically categorized
Prolaris	Prognostic; Biopsy tissue	46-gene RNA signature to predict PCa mortality	CLIA-validated (2010)	Predicts BCR and metastasis. Guides AS vs. treatment [35].	Requires sufficient tumor tissue. Limited validation in diverse populations. NCCN: Not specifically categorized
ProMark	Prognostic; Biopsy tissue	Protein-based assay (8 biomarkers) to stratify risk from biopsy tissue	CLIA-validated (2017)	Distinguishes indolent vs. aggressive disease. Works with small samples [36].	Less widely adopted. Limited long-term outcome data. NCCN: Not specifically categorized
Decipher	Prognostic; Biopsy tissue (RNA biomarker test with 22 genes)	Predicts 5-/10-year metastasis risk following radical prostatectomy/RT. Guides adjuvant therapy.	CLIA-validated (2015)	Identified men with favorable- intermediate risk disease with adverse pathology [37].	Requires biopsy or prostatectomy tissue; cost; limited pre-treatment applicability. NCCN: Category 2A with caveat *
CAPRA (Cancer of the Prostate Risk Assessment) Score	Prognostic; Clinical parameters (no biological specimen)	Clinical nomogram using PSA, stage, grade, etc.	N/A (Clinical tool)	Pre-treatment risk stratification at diagnosis [38].	Relies on clinicopathologic variables; does not directly assess tumor biology. NCCN: Not specifically categorized
**C. Genomic/Predictive Biomarkers for Targeted Therapy**					
**Biomarker**	**Diagnostic or Prognostic;** **Sample Type**	**Main Purpose**	**Year of FDA Approval (If Applicable)**	**Indication/Clinical Use**	**Key Limitation(s); Evidence Level (Based on NCCN Recommendation)**
HRR Gene Mutation (e.g., BRCA1/2, ATM)	Predictive Tumor tissue (NGS); blood (germline testing); plasma (ctDNA)	Identifies homologous recombination deficiency (HRD) for PARP inhibitor sensitivity.	2020	Metastatic PCa to guide PARP inhibitor therapy.	Not all alterations confer equal therapeutic sensitivity; assay and panel variability. [39]; NCCN: Category 1
MSI-H/dMMR (Microsatellite Instability-High/Mismatch Repair Deficient)	Predictive Tumor tissue (NGS, IHC, PCR); ctDNA (selected platforms)	Identifies tumors with high mutational burden for immune checkpoint inhibitor response.	2017	Advanced, treatment-refractory tumors for immunotherapy.	Rare in PCa (<5%); requires tumor tissue or comprehensive genomic profiling [40]; NCCN: Category 2A
TMB-H (High Tumor Mutational Burden	Predictive Tumor tissue (NGS, IHC, PCR); ctDNA (selected platforms)	Quantitative measure of mutations; surrogate for potential immunotherapy benefit.	2020	Alternative biomarker for immunotherapy consideration.	Lack of prostate cancer–specific cut-offs; often requires concurrent MSI assessment [41]; NCCN: Category 2A
PTEN Loss	Predictive Tumor tissue (IHC, FISH, NGS)	Tumor suppressor loss; associated with PI3K/AKT pathway activation and worse prognosis.	None	Emerging trials show that it is a prognostic and potentially predictive marker for AKT inhibitors.	Not currently a standalone therapy-guiding biomarker; typically requires integration with broader genomic context. [42,43,44]; NCCN: Category 2A

Footnote: NCCN (National Comprehensive Cancer Network) Categories of Evidence and Consensus: Category 1—Based upon high-level evidence (≥1 randomized Phase III clinical trial or high-quality, robust meta-analyses), there is uniform NCCN consensus (≥85% panel support) that the intervention is appropriate. Category 2A—Based upon lower-level evidence, there is uniform NCCN consensus (≥85% panel support) that the intervention is appropriate. Category 2B—Based upon lower-level evidence, there is NCCN consensus (≥50% but <85% panel support) that the intervention is appropriate. Category 3—Based upon any level of evidence, there is major NCCN disagreement that the intervention is appropriate. All NCCN recommendations are Category 2A unless otherwise indicated. * Caveat: In the absence of prospective trials, caution is warranted if using these prognostic tools to influence treatment decisions. The Panel awaits future trials that confirm the initial results described here.

**Table 2:** Galectin-3 inhibition across various malignancies.

Cancer Type	Gal-3’s Pathophysiological Role	Therapeutic Agent	Class of Drug	Key Findings & Implications
Melanoma & HNSCC	Immune Escape: Gal-3 acts as a negative immune checkpoint, blocking anti-PD-1 antibody binding to T-cells.	Belapectin + Pembrolizumab	Small molecule Galectin-3 inhibitor	Phase I study showed objective response rates of 50% (melanoma) and 33% (HNSCC). Demonstrates that Gal-3 inhibitors may enhance immunotherapy by reversing immune suppression [94,95].
Chronic Myelogenous Leukemia (CML)	Drug Resistance: Gal-3 confers multidrug resistance by promoting the activity of drug efflux pumps.	NA	Gal-3 Antagonists	Preclinical studies show Gal-3 inhibition can overcome resistance to TKIs and genotoxic agents, re-sensitizing tumors to chemotherapy [91].
Lung & Pancreatic Cancer	KRAS Addiction: Gal-3 supports pro-survival pathways in KRAS-driven tumors.	GB1107	Novel small molecule Galectin-3 inhibitor	Identified as a druggable vulnerability in hard-to-treat tumors, showing Gal-3 as a key mediator of oncogenic signaling beyond the AR pathway [96].
Advanced Solid Tumors	Broad Pro-tumorigenic Effects: Involved in tumor growth, metastasis, and angiogenesis.	GCS-100 (MCP)	Modified citrus pectin (MCP)	Phase I study showed good tolerability; stable disease in 16 of 24 patients, providing initial evidence of anti-tumor activity and safety [97].
Recurrent PCa	Slowing disease progression and metastasis.	Pecta Sol	Modified citrus pectin (MCP)	Phase II pilot study found MCP lengthened PSA doubling time in 70% of patients, suggesting potential to slow disease progression [79].

## Data Availability

No new data were created or analyzed in this study. Data sharing is not applicable to this article.
